# Attitudes and Behavior of Patients with Gynecologic Malignancy Towards Sexual Issues: a Single-institutional Survey

**DOI:** 10.1007/s13187-019-01653-9

**Published:** 2019-12-05

**Authors:** Wei Chen, Qin Ma, Xiaoqin Chen, Chenyan Wang, Huijuan Yang, Yi Zhang, Shuang Ye

**Affiliations:** 1 Department of Gynecologic Oncology, Fudan University Shanghai Cancer Center, Shanghai, China; 2 Department of Obstetrics and Gynecology, Minhang Hospital, Fudan University, the Central Hospital of Minhang District, Shanghai, China; 3 Department of Oncology, Shanghai Medical College, Fudan University, Shanghai, 200032 China

**Keywords:** Gynecologic cancer, Sexuality, Questionnaire, Survey

## Abstract

To better understand patients’ perspectives and preferences for sexual issues and to provide information to guide medical staff in delivering sexual health care, an anonymous, cross-sectional survey of inpatient gynecologic cancer patients was conducted from April 2017 to March 2018. The questionnaire consisted of three parts: basic information, a history of sexuality discussion, and eight preference questions. A total of 1192 patients were included, and the median age was 43 years. More than half of the patients had a junior high school education or less and low income. Of the patients, 46.2% agreed that, “Doctors should deal with patients’ sexual issues,” and 56.2% agreed that, “Doctors should raise the topic of sexual issues.” While 69.1% of the patients were willing to raise the topic themselves if any sexual problem existed, 35.9% of the patients agreed, “It’s not the right time to discuss sex due to my severe disease.” Less than 30% of the patients thought that “There’s no need to intervene because sex is private” and “I do not feel comfortable discussing sex.” A total of 41.1% of the patients were interested in undertaking basic sexual health consultation. Multiple logistic regression analysis demonstrated the following predictive factors for a history of consultation: young age; good education; and agreement with the statements, “Doctors should raise the topic of sexual issues,” “If any sexual problem exists, I will raise the topic,” and “I have an interest in participating in sex-counseling activities.” Patients were willing to discuss sexual issues and were interested in sexual health training. It is important to increase the medical staff awareness of the need to inquire about and address patients’ sexual issues.

## Introduction

Sexuality is now regarded as an important component of quality of life in cancer survivors, especially for patients with a gynecologic malignancy. However, both radical surgery and radiotherapy have a great influence on patients’ sexuality, intimacy, and sexual function [1, 2]. Across multiple datasets, the incidence of sexual dysfunction ranges from 30 to 90% among female cancer survivors [3, 4]. At the same time, patients with gynecologic malignancies are more likely to have mental stress and barriers than patients with other kinds of tumors [5, 6]. Because of the particularity and concealment of sexual issues, patients are either not willing to seek help or do not know how to seek help [7]. It has been found that 42% to 74% of women with a history of gynecologic or breast cancer were interested in receiving professional help [6, 8]. However, only 7% to 40% of the survivors actually sought or received such help [6, 8]. Therefore, it is of crucial significance to better understand the experiences, needs, and preferences of patients with gynecologic cancer regarding sexuality and sexual health care.

In this study, we investigated the behaviors and attitudes of patients with gynecologic cancer towards sexuality consultations in the clinical setting to reveal the correlative factors of the likelihood of participating in consultations regarding sexual issues.

## Methods

**Fig. 1:**
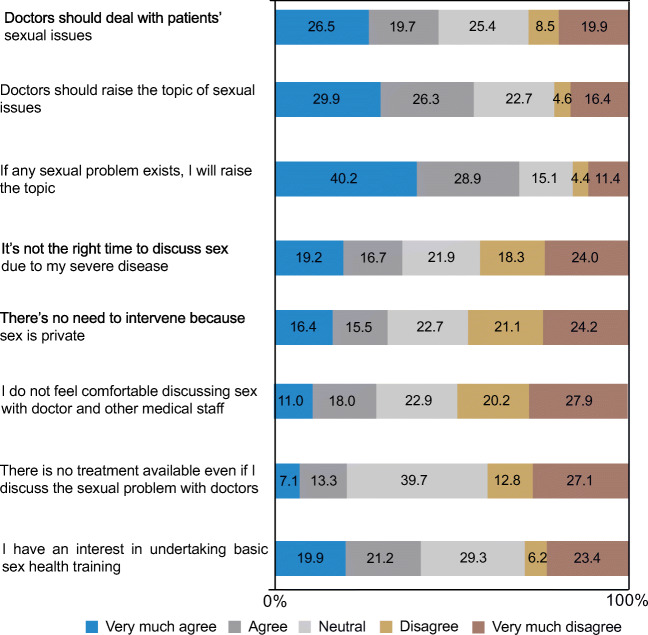
Respondents’ attitudes toward eight statements regarding sexuality and cancer (*N* = 1192)

After obtaining approval from the institutional review board, we conducted a cross-sectional questionnaire-based study of women who were admitted to our department for gynecologic cancer operations between April 2017 and September 2018. Patients were required to be able to read and write in Chinese to be included in the study. An anonymous self-reported questionnaire was given by an experienced nurse to the participants during their postoperative hospital stay. The questionnaire was adapted and revised from a previous publication assessing the attitudes and practices of breast cancer surgeons regarding sexual issues [9]. It consisted of three parts: basic demographic information (age, marital status, occupation, monthly income, and education), previous experience of receiving consultation on sexual-related issues, and eight preference questions (specific details are listed in Fig. 1, Table 3).

The Statistical Package for Social Science (SPSS) (Version 20.0, SPSS, Inc., Chicago, IL, USA) was used for the analyses. Demographic information was presented using descriptive statistics. The associations between the patients’ personal information (demographic characteristics and sexuality consultation history) and attitudes were evaluated by the chi-square test and analysis of variance as appropriate. The significant variables were further incorporated into multiple logistic regression analysis to identify the predictive factors for sexuality consultation. All *P* values reported were two tailed, and *P* < 0.05 was considered statistically significant.

## Results

### Demographic information and sexuality consultation experiences

Table 1 presents the demographic characteristics of the 1192 participants. The median age was 43 years (range, 18–69 years). Most participants were married (96.2%) and parous (91.8%). More than half of the respondents were of a poor education level (56.4% with secondary school and under) and low income (51.9% with less than 3000 yuan/month). In total, 80 patients (6.7%) reported once discussing sexuality: 64 with doctors (80.0%), nine with nurses (11.3%), two with both doctors and nurses (2.5%), two with other health care workers (2.5%), and three patients (3.8%) did not specify.

**Table 1:** Associations between demographic information and consulting experiences

Characteristic	Total (*n* = 1192)	Once consulted (*n* = 80)	Not consulted (*n* = 1112)	*P* value
Age (year), median (range)	43 (18–69)	40 (25–53)	43 (18–69)	< 0.001*
Relationship status, *n* (%)				
Single	44 (3.7)	1 (1.3)	43 (3.9)	0.356#
Married	1147 (96.2)	79 (98.8)	1068 (96.0)	
Divorced	1 (0.1)	0	1 (0.1)	
Gravidity, *n* (%)				
Nulliparous	98 (8.2)	6 (7.5)	92 (8.3)	0.841#
Porous	1094 (91.8)	74 (92.5)	1020 (91.7)	
Monthly income (yuan), *n* (%)				
< 3000	619 (51.9)	29 (36.3)	590 (53.1)	*0.012#*
3000~8000	466 (39.1)	40 (50.0)	426 (38.3)	
> 8000	107 (9.0)	11 (13.8)	96 (8.6)	
Education background, *n* (%)				
Secondary school and under	672 (56.4)	32 (40.0)	640 (57.6)	*< 0.001#*
High school	213 (17.9)	9 (11.3)	204 (18.3)	
University and higher	307 (25.6)	39 (48.8)	268 (24.1)	

*P* values with statistical significance were italicized

*One-way analysis of variance

^#^Chi-square test

Patients were divided into two groups based on their consultation experiences (Table 1). Not surprisingly, younger patients were more likely to discuss sexuality with the medical staff (*P* < 0.001). Again, patients with high income (*P* = 0.012) and a good education (*P* < 0.001) tended to initiate discussions themselves.

Among the patients with a consultation history, 69 offered specific counseling contents, which led to a total of 78 questions listed in Table 2. The patients were mostly concerned about the timing of resuming sex after surgery (38.5%), followed by the issues of intercourse bleeding (16.7%).

**Table 2:** Most frequent subjects of consultations relating to sexual issues (in descending order)

Rank no.	Topic	No. of patients	%
1	Timing for resuming sex	30	38.5
2	Bleeding after intercourse	13	16.7
3	Safety of having sex (is it okay to have sex?)	11	14.1
4	Points to note when having sex	10	12.8
5	Safety of future pregnancy	5	6.4
6	The impact of surgery on sex	4	5.1
7	Frequency of sex	3	3.8
8	The relationship between cancer and sex	2	2.6
9	Will having sex cause relapse or affect prognosis?	1	1.3

### Attitudes towards sexuality discussion

Figure 1 shows the respondents’ attitudes of discussing sexual issues in clinical settings as indicated in their answers to eight statements. Regarding the statement indicating that doctors should deal with patients’ sexual issues, 46.2% of patients agreed, while 28.4% disagreed. A total of 56.2% of the respondents agreed that doctors should raise the topic of sexual issues. On the other hand, 69.1% of patients expressed that they would raise the topic if any sexual problem exists. Only one-third of the patients thought that it was not the right time to discuss sex due to their severe disease. Nearly 32% of the respondents considered there was no need to intervene because of the privacy of sexual issues. In addition, 29% of the respondents felt uncomfortable discussing sex with doctors or other medical staff. Approximately 41% of the patients showed interest in participating in sex-counseling activities.

As clearly seen from Table 3, patients with or without a consultation history had apparently different attitudes towards sexuality discussion. Patients who once consulted had a much higher tendency to agree that “Doctors should deal with patients’ sexual issues” and “Doctors should raise the topic of sexual issues.” Significantly fewer patients in the consultation group disagreed that “If any sexual problem exists, I will raise the topic.” Concerning the statement “I do not feel comfortable discussing sex with doctor and other medical staff,” 55.0% and 47.7% of those who once consulted or never consulted disagreed, respectively. As expected, participants with a past consultation history reported greater interest in participating in sex-counseling activities (*P* < 0.001).

**Table 3:** Patients’ attitudes toward sexuality based on consultation experiences

Questions	Attitudes	Once consulted (%)	Never consulted (%)	*P* value#
Doctors should deal with patients’ sexual issues.	Agree	68.8	44.6	*< 0.001*
	Disagree	10.0	29.7	
	Neutral	21.3	25.7	
Doctors should raise the topic of sexual issues.	Agree	82.5	54.4	*< 0.001*
	Disagree	2.5	22.3	
	Neutral	15.0	23.3	
If any sexual problem exists, I will raise the topic.	Agree	87.5	67.7	*0.001*
	Disagree	2.5	16.8	
	Neutral	10.0	15.5	
It’s not the right time to discuss sex due to my severe disease.	Agree	28.8	36.5	0.218
	Disagree	42.5	42.3	
	Neutral	28.8	21.4	
There’s no need to intervene because sex is private.	Agree	28.8	32.2	0.564
	Disagree	43.8	45.5	
	Neutral	27.5	22.3	
I do not feel comfortable discussing sex with doctor and other medical staff.	Agree	21.3	29.5	0.266
	Disagree	55.0	47.7	
	Neutral	23.8	22.8	
There is no treatment available even if I discuss the sexual problem with doctors.	Agree	23.8	20.2	0.493
	Disagree	33.8	39.5	
	Neutral	42.5	40.3	
I have an interest in taking sex-counseling activities.	Agree	60.0	39.8	*< 0.001*
	Disagree	8.8	31.1	
	Neutral	31.3	29.1	

*P* values with statistical significance were italicized

^#^Chi-square test

### Correlative factors for initiating discussions of sexual issues

Lastly, we evaluated the predictive factors for a history of sexuality consultation by incorporating all the significant variables in the previous univariate analysis into a multiple logistic regression analysis (Table 4). We found that younger patients (*P* = 0.037) with a higher educational background (*P* = 0.004) were more likely to initiate discussions. Interestingly, respondents who agreed that “Doctors should raise the topic of sexual issues” were more likely to consult about sex issues with their doctors (*P* < 0.001). In terms of attitudes towards the statements “If any sexual problem exists, I will raise the topic” and “I have an interest in taking sex-counseling activities,” the two groups with and without a consulting history were different.

**Table 4:** Correlative factors for a history of consultation about sexual issues

Variables	*P* value	OR	95%CI
Age	*0.037*	1.032	1.002–1.063
Education	*0.004*	0.668	0.506–0.882
Income	0.691	/	/
Doctors should deal with patients’ sexual issues	0.227	/	/
Doctors should raise the topic of sexual issues	*< 0.001*	2.378	1.472–3.840
If any sexual problem exists, I will raise the topic	*0.024*	1.837	1.085–3.108
I have an interest in taking sex-counseling activities.	*0.022*	1.501	1.060–2.127

*P* values with statistical significance were italicized

*OR*, odds ratio; *CI*, confidence interval

## Discussion

Sexual issues remain unaddressed for many cancer survivors, particularly women [10]. This phenomenon is also very significant in China. Recently, a descriptive correlational study of 156 Chinese patients with gynecologic cancer revealed that sexual dysfunction was an important concern (62.2%), and the rate of sexual inactivity was 70.5% [11]. Despite this, little attention has been paid to the behaviors and attitudes of sexual issues in survivors with gynecologic malignancy.

In our survey of 1192 Chinese gynecologic cancer patients, less than 7% of respondents had consulted health care professionals about sexual problems, although only approximately one-third of the patients held a negative attitude towards the discussion of sexual topics. There might be various reasons for this phenomenon. First, most patients agreed that doctors should initiate discussions about sexual issues. Patients often believe that the provider would raise the issue if it was important, suggesting they may be reluctant to initiate the topic because they feel that they are burdening their provider [5, 12]. Second, most patients may not have sex after their diagnosis or treatment. In our survey, nearly 70% of patients indicated a willingness to initiate a discussion with their doctor when they had sexual problems. However, we cannot assume that these patients had no problems if they did not ask questions. According to two studies from China, 56~71% of participants did not engage in sexual intercourse after treatment [11, 13]. There are many concerns about resuming sexual activity, which may be the current situation of many gynecological malignancy survivors in China. The list of important topics for patients related to counseling in our study also provides similar evidence: 38.5% of the questions were related to the timing of resuming sex after surgery, and 14.1% were about the safety of having sex. Third, a large percentage of respondents did not believe they could receive treatment from an oncologist for sexual issues. As shown by our survey, 20% of the patients expressed a negative attitude, and nearly 40% were noncommittal about whether they could receive available treatment for sexual problems from their doctors. Previous studies by Beckjord et al. found that the presence of emotional distress, as well as treatment, could have a significant impact on the sexual quality of life [14]. Although much of this work has been done in breast cancer survivors [15–18], emerging literature shows similar figures for the survivors of gynecologic cancer affected [1, 19, 20]. Patients may experience problems that involve mental and emotional changes that they do not think oncologists can solve for them. This also limits patients from initiating discussions about sexual issues during the limited visit time. Finally, a poor educational background might be another barrier, according to our survey. The reason may be that patients with a low education, who represent the majority of the respondents, have less knowledge of sexual health and do not know how to raise the topic. Thus, relying on the patients to raise sexual concerns is an ineffective strategy because patients often do not ask for help, even if they show interests in receiving care.

From the healthcare providers’ perspective, they also do not routinely discuss sexual concerns during follow-up [21]. A nationwide survey of Dutch surgical oncologists found that counselling on sexual function was performed by only 9.2% of the surgeons [22]. Similarly, approximately 7.9% of Swiss gynecologists routinely discussed sexual issues with their patients [23]. A number of studies have depicted some possible barriers: a lack of appropriate training, embarrassment, time pressures, unfamiliarity with treatment options, and prioritizing other physical symptoms [12, 24, 25]. There has been no large-scale study of providers’ attitudes in China, and some small studies suggest similar results [26].

To provide patient-centered, cost-effective, and time-efficient care, providers should identify patients who desire discussions about sexuality or who need help and learn how and when to best deliver this information. First, to facilitate patients’ awareness of sexual issues and to initiate effective discussions, doctors need to be encouraged to include information on treatment-induced sexual changes as a part of routine follow-up visits. Second, there is a need to tailor the way of providing information based on individuals. Stabile et al. found that patients of all ages preferred to review and discuss written information with their medical team. Older women preferred to read material on their own (*P* = 0.012), whereas younger women wanted to discuss these materials directly with the medical team (*P* < 0.017). Younger women reported more interest in the online intervention modality (*P* < 0.001) [27]. Our data suggest that more than 41% of respondents indicated interest in undertaking sex-counseling activities. Del Pup et al. [28] suggested that providers should offer the patients resources that could be used to preserve or improve their sexual function or knowledge if there was no proper time or place for face-to-face counseling. Advice and training can be provided in a variety of ways; for example, providing patients with information brochures, online tweets, and apps may be good choices. In particular, these methods could provide basic sexual health knowledge and are not limited by time and space conditions. The other possible approach for addressing sexual health within the limited visit time is to have the conversation started by trained nurses [11] or carry out a questionnaire survey of sexual situation before counseling [28].

Our study has a number of limitations. First, it only included patients at a single academic cancer center and thus may have limited applicability to other environments and locations. Second, given that the study is a cross-sectional survey, we cannot conclude a causal relationship between the attitudes or behaviors of sexual consultation and the correlative factors.

## Conclusions

Sexual issues are an important part of quality of life for both cancer survivors and their caregivers; however, they remain largely underrecognized and unaddressed for various reasons. Relying on patients to raise sexual concerns is an ineffective strategy because patients often do not ask for help, even if they report an interest in receiving care. Most importantly, healthcare providers should be encouraged to take the initiative to initiate the discussion through basic training or standard patient simulation. In addition, it is important for healthcare workers to identify patients who desire sexuality discussions. For example, age, education, and the idea that doctors should initiate these discussions are also relevant factors. Moreover, different kinds of sexual discussions could be designed and tailored to individuals, including face-to-face conversations, reading materials, online modalities, and sexual-counseling activities. We are now trying to write some materials based on the current study. Last but not least, it is important for doctors to address the sexual issues of patients with gynecologic malignancy in follow-up visits.
